# An integrated model of rapid torque production: neuromuscular adaptations under fatigue after strength training

**DOI:** 10.1007/s00421-026-06321-7

**Published:** 2026-07-02

**Authors:** Leonardo Henrique Perinotto Abdalla, Benedito Sérgio Denadai, Natália de Menezes Bassan, Ryan Michael Broxterman, Camila Coelho Greco

**Affiliations:** 1 Human Performance Laboratory, UNESP, Av. 24 A, 1515, Bela Vista, Rio Claro, S.P CEP – 13506-900 Brazil; 2 Department of Internal Medicine, University of Utah, Salt Lake City, UT USA; 3 Geriatric Research, Education, and Clinical Center, VAMC, Salt Lake City , UT USA

**Keywords:** Maximal strength training, Rate of torque development, Critical RTD, Impulse

## Abstract

This study investigated the effects of short-term maximal strength training on the dynamics of maximal torque and torque impulse, assessed in non-fatigued and fatigued muscles across distinct temporal domains. Nineteen healthy men participated: ten performed a unilateral short-term training program of the knee extensors, while nine maintained their habitual routines as a control group. Before and after the intervention, all participants performed a 5-minute all-out test. The test was used to characterize the torque–time hyperbolic relationship and to determine critical torque (CT) and critical impulse (CI), as well as the accumulated impulse above CT and CI (iCT′ and iCI′). In the non-fatigued condition, training significantly increased maximal torque (*p* = 0.040), with no changes in torque impulse or integrated electromyography (iEMG) across the 30, 100, and 200 ms time windows (all *p* > 0.05). During the 5-min all-out test, training significantly increased the time constant (τ) of the decay in maximal torque and total impulse of torque (all, *p* < 0.05), while CT and CI remained unchanged (all, *p* > 0.05). In parallel, iCT′ and iCI′ increased significantly (all, *p* < 0.05). Analysis of iEMG revealed an increase in the intercept of the iEMG–contraction relationship (*p* < 0.05), with no change in its slope (*p* > 0.05). These findings demonstrate that short-term maximal strength training enhances maximal torque and fatigue tolerance during sustained maximal efforts via slower neuromechanical decay and greater impulse availability above critical thresholds, without altering the critical limits of torque or impulse.

**Supplementary Information:**

The online version contains supplementary material available at https://doi.org/10.1007/s00421-026-06321-7.

## Introduction

The rate of torque development (RTD), defined as the slope of the torque–time curve, is widely used to characterize the rapid production of torque (Aagaard et al. 2002). RTD can be assessed within discrete time windows from the onset of contraction (Maffiuletti et al. 2016), allowing for the differentiation between physiological determinants predominantly associated with neural activation (< 100 ms) and those related to muscular properties and neuromuscular fatigue (> 100 ms) (Folland et al. 2014; D’Emanuele et al. 2021).

Improvements in RTD are fundamentally attributed to two neural mechanisms: increase in motor unit recruitment and/or elevation of the initial firing rate (Aagaard et al. 2002; Enoka and Duchateau 2017). For example, Aagaard et al. (2002) observed that strength training increased the initial neural drive, resulting in greater RTD up to 200 ms. Consistent with this principle, Del Vecchio et al. (2023) reported that, although four weeks of explosive training, increased maximal torque and sustained firing rate (300 ms), no changes occurred in motor unit recruitment or firing rate up to 200 ms, which explains the absence of modifications in RTD. This discrepancy in the training-induced effects on RTD underscores the specificity of the neural adaptations that underpin it, particularly those associated with the modulation of initial neural drive (Maffiuletti et al. 2016).

Although RTD is widely recognized for its functional relevance, particularly in training and performance contexts, most mechanistic evidence comes from measurements performed in non-fatigued muscles, using only the slope of the torque–time curve within discrete time windows (D’Emanuele et al. 2021). This methodological limitation creates a conceptual constraint, as it reduces the ecological validity of the findings and prevents understanding of RTD behavior during progressive fatigue development, which is crucial for elucidating training adaptations and performance under maximal effort conditions (Maffiuletti et al. 2016; D’Emanuele et al. 2021). Fatigue during exercise is usually defined as a reversible decline in maximal muscle force or torque-generating capacity (Gandevia 2001).

In a recent study published by our group (Abdalla et al. 2025), the Critical Power (CP) model was applied to a 5-min all-out isometric test (Burnley 2009) to investigate the behavior of RTD under fatigue. In this model, CP represents the threshold for sustainable performance, while the W′ reflects work capacity above this threshold, limited by peripheral fatigue (Burnley et al. 2012; Broxterman et al. 2017). We observed that RTD exhibited a hyperbolic profile similar to torque, reaching an asymptotic value termed “Critical RTD” at the end of the test, suggesting parallel modulation by fatigue-related mechanisms. However, the methodology employed (slope of the torque–time curve) did not allow integration of the data to quantify rapid torque production above this limit, which could be captured by calculating the impulse, defined as the area under the torque–time curve.

Recent evidence indicates that W′ exhibits plasticity (Sawyer et al. 2014; Bassan et al. 2019), suggesting that analogous functional reserves may be modulated through training–induced adaptations. For example, Bassan et al. (2019) reported an increase in W′ after three weeks of maximal strength training, with no change in CP, attributing this effect to an enhanced tolerance to peripheral fatigue, possibly mediated by group III/IV afferent feedback. Complementarily, Siegler et al. (2013) demonstrated that reducing peripheral metabolic stress through bicarbonate supplementation increased RTD under fatigue, indicating that peripheral signals can modulate neuromuscular drive during high–intensity contractions. Considering that these processes, encompassing peripheral fatigue, contractile limitations, and sensorimotor modulation, influence both RTD (Siegler et al. 2013, 2015, 2016) and W’ (Bassan et al. 2019), it is plausible to propose that the accumulated impulse above the Critical RTD (analogous to W′) may also display training-induced plasticity.

To overcome these methodological and conceptual limitations, the present study proposed analyzing the impulse associated with RTD in discrete time windows during a 5-min all-out isometric test. This approach allowed for the estimation of the Critical Impulse (CI, analogous to Critical RTD) and the impulse accumulated above CI (iCI′). The primary aim was to investigate the effects of strength training on maximal torque and impulse in non-fatigued muscles and under progressive fatigue. Our hypotheses were that: (i) training would increase maximal torque in non-fatigued muscle, but would not significantly alter impulse or neural drive up to 200 ms; (ii) during the all-out test, the trained group would demonstrate a greater capacity to sustain maximal torque and rapidly produce torque under fatigue via a slower decline in impulse and smaller reductions in maximal torque; and (iii) training would increase the iCI′ without affecting the CI, mirroring the pattern observed for W′ and indicating enhanced fatigue tolerance.

## Methods

### Participants and ethical considerations

Nineteen healthy, physically active young men (mean ± SD: 25.1 ± 4.1 years; 74.7 ± 16.1 kg; 175.5 ± 6.8 cm) voluntarily participated in this study. Inclusion criteria were: (i) male sex; (ii) age between 18 and 35 years; (iii) no systematic resistance training in the 6 months prior to data collection; and (iv) being physically active but not engaged in structured training programs. Exclusion criteria included: (i) history of lower-limb musculoskeletal injury or knee pain; (ii) neurological or cardiovascular disorders; (iii) use of ergogenic or pharmacological substances known to affect neuromuscular performance; and (iv) inability to complete all experimental sessions. All participants were fully informed about the study objectives, procedures, and potential risks, and provided written informed consent prior to participation. The study was conducted in accordance with the ethical principles of the Declaration of Helsinki. The experimental protocol was approved by the institutional Research Ethics Committee (CAAE: 58598916.0.0000.5465).

### Experimental design

The study was conducted in a temperature-controlled laboratory (21–23 °C) and at consistent times of day. Participants attended twelve sessions: three testing sessions (two pre- and one post-intervention) and nine intervention sessions. During the first visit, participants were familiarized with the dynamometer, and positioning settings were recorded for replication. In the second visit, they performed three maximal voluntary contractions (MVC) followed by a 5-min all-out test, after which they were randomly allocated to either the control or training group. Following the intervention period, participants completed a post-intervention 5-min all-out test. Participants were instructed to arrive rested, adequately hydrated, and to avoid strenuous exercise prior to each session.

### Procedures

#### Isokinetic dynamometer

The tests were performed using an isokinetic dynamometer (Biodex System 3, Shirley, NY, USA). For knee extension assessment, participants were seated with the hip and knee joint angles set at 85° and 75°, respectively, and were stabilized to minimize trunk movement. The dynamometer axis was aligned with the flexion–extension axis of the right knee, and the lever arm was secured with a non-elastic strap. Chair settings were recorded during the first session and replicated thereafter to reduce system compliance (Maffiuletti et al. 2016). Participants relaxed the limb to allow gravitational correction, and all tests were performed on the dominant leg.

#### All-out test

The hyperbolic relationship between torque impulse and time, as described by the Critical Power (CP) model (Monod and Scherrer 1965), was assessed using a validated 5-min all-out test for the knee extensors (Abdalla et al. 2025). The protocol consisted of 60 MVC (3 s contraction, 2 s rest). Prior to the test, participants performed five submaximal contractions followed by 5 min of rest, and then three 3-s MVC with 3-min intervals to determine maximal torque in the non-fatigued condition; the highest value was adopted as the reference. Participants were informed of their MVC value and encouraged to match or exceed it during the first three contractions. During the test, they were instructed to contract “as fast and as forcefully as possible,” received real-time torque feedback, but remained blinded to the elapsed time and number of remaining contractions to minimize pacing.

#### Intervention protocol

Participants in the control group did not perform any intervention and were instructed to maintain their habitual physical activity. The training consisted of unilateral maximal torque exercise for 3 weeks (2 sessions per week), with three sets of ten 4-s isometric MVC (2-s intervals; 2-min between sets) on the dominant limb.

### Measurements and data analysis

#### Isokinetic dynamometer

The isokinetic dynamometer (Biodex System 3^®^, Shirley, NY, USA) was synchronized with a biological signal acquisition system (Miotec^®^, Porto Alegre, RS, Brazil). Torque signals were sampled at 2000 Hz to prevent aliasing and ensure data fidelity, according to the Nyquist criterion. Resting torque noise was ~ 0.74 Nm (~ 0.25% MVC) (Maffiuletti et al. 2016). The torque curves were smoothed by a digital fourth-order zero-lag Butterworth filter with a cutoff frequency of 40 Hz (Winter 2009). Maximal torque was defined as the greatest instantaneous torque. Critical torque (CT; analogous to CP) was defined as the average of the last six contractions, and the (iCT’) was calculated as the cumulative impulse above the CT (Burnley et al. 2009).

#### Torque impulse

Torque impulse during the initial phase of contraction was assessed according to the method proposed by Aagaard et al. (2002) and Maffiuletti et al. (2016). Three-time windows were analyzed: 0–30 ms, 0–100 ms, and 0–200 ms. Torque impulse was calculated as the integral of the torque-time curve within each interval, with the onset of contraction defined as the first point of the smoothed torque curve exceeding 1 Nm (Aagaard et al. 2002; Maffiuletti et al. 2016). A MATLAB^®^ R2017a routine (MathWorks Inc., Natick, MA, USA) was used to automatically detect this point. The detection was then visually validated by the operator, who carefully reviewed the torque curve to ensure the accuracy of the onset detection, thereby enhancing the reliability of the measurement and minimizing potential interpretation errors.

#### Critical impulse (CI) and impulse accumulated above CI (iCI′)

Analyses were performed using time windows of 30, 100, and 200 ms. For each contraction, torque impulse was calculated as the integral of the torque–time curve within the respective time window, and total impulse was obtained by summing the impulses of all contractions. Critical Impulse (CI) was defined as the mean impulse of the last six contractions during the 5-min all-out test, analogous to Critical RTD. The impulse accumulated above CI (iCI′) was calculated as the impulse generated above CI, analogous to W′.

#### Electromyography (EMG)

Surface EMG was recorded from the vastus lateralis using bipolar Ag–AgCl electrodes (2 cm inter-electrode distance) following SENIAM (Surface Electromyography for the Non-Invasive Assessment of Muscles) recommendations, with a Miotec^®^ device (Porto Alegre, RS, Brazil). Skin was prepared to minimize impedance, and a reference electrode was placed over the ulnar styloid (Hermens 2000). Resting electrical activity was maintained below 5 µV prior to data acquisition. For signal processing, a custom routine developed in MATLAB^®^ was used, in which raw signals were band-pass filtered between 20 and 499 Hz using a second-order Butterworth filter and subsequently processed with the filtfilt function, ensuring zero-phase filtering and preservation of the temporal characteristics of the signal (Maffiuletti et al. 2016). Based on the filtered signals, the onset of muscle activation was determined for each of the 60 contractions of the all-out test by automatically identifying the first point at which the EMG signal exceeded a threshold corresponding to three standard deviations above the resting baseline (Hodges et al., 1996; Staude et al. 2001). Following automatic detection, the identified onset was visually inspected and confirmed by the operator, reducing assessor-dependent variability, and enhancing the reliability of the procedure (Staude et al. 2001). This criterion (3 SD) differs from that adopted for the torque signal (1 Nm), reflecting the inherent temporal differences between electrical and mechanical responses. EMG signals were analyzed over three specific time windows (30, 100, and 200 ms) relative to EMG onset, corresponding to the same intervals used for torque impulse. Within each window, electromyographic activity was quantified using integrated EMG (iEMG), allowing the assessment of cumulative electrical activity during the early phase of contraction and ensuring methodological consistency with the torque impulse analysis. Neural drive during the all-out test was characterized using linear regression of iEMG across contractions, yielding intercept and slope values. Although EMG recordings from a single muscle do not capture the full neuromuscular coordination of the quadriceps group, the vastus lateralis was selected due to its widespread use in the literature and its ability to provide a reliable and representative estimate of quadriceps activation during knee extension tasks. Previous studies have demonstrated consistent and robust activation patterns of the vastus lateralis under isometric and fatiguing conditions (Masuda et al. 1999; Pincivero and Coelho 2000; Alkner et al. 2000; Bilodeau et al. 2003), although it is acknowledged that individual muscles within the quadriceps may exhibit task-dependent activation differences (Hug and Tucker 2017; Farina et al. 2014).

#### Time constant (***τ***)

The time constant (τ) of the exponential decay was calculated for maximal torque and total impulse of torque (30, 100 and 200 ms) during the 5-min all-out test, using an exponential model with a plateau. τ represents the time required for maximal torque or total impulse of torque to decay to approximately 37% of the difference between its initial value and the asymptote.

#### Statistical analyses

An a priori power analysis was performed using GPower (version 3.1.9.7) for a repeated-measures ANOVA (Group × Time interaction), based on the primary outcome (peak torque). A moderate-to-large effect size was assumed (f = 0.33; Cohen, 1988), with α = 0.05 and power (1 − β) = 0.80. Assuming two groups, two time points, and a correlation of 0.50 between repeated measures, the required total sample size was estimated at *n* = 20.

Data are presented as mean ± standard deviation (SD) and 95% confidence intervals (95% CI). Depending on the nature of the outcome variable, different statistical models were applied. For delta (Δ) variables, repeated-measures ANOVA was employed. Normality of distributions was verified using the Shapiro–Wilk test, while sphericity was assessed using Mauchly’s test; when violated, the Greenhouse–Geisser correction was applied. For the non-fatigued muscle condition, ΔMaximal Torque and ΔTorque Impulse were analyzed using a 2 × 4 factorial design, whereas Δintegral of initial electromyographic activity (ΔiEMG) was analyzed using a 2 × 3 design. During the all-out test, Δτ (time constant) for maximal torque and total torque impulse, as well as ΔCT, ΔCI, ΔiCT′, and ΔiCI′, were analyzed using a 2 × 4 factorial design. The Δintercept and slope of the iEMG–time relationship, as well as normalized variables (iCI′ adjusted for the iEMG intercept), were analyzed using 2 × 3 factorial designs. Significant main effects and interactions were followed by Tukey’s HSD post hoc test. Effect sizes are reported as partial eta squared (ηp²), interpreted as small (≤ 0.06), moderate (0.07–0.14), and large (> 0.14). Absolute (pre- vs. post-intervention) data were analyzed using a linear mixed-effects model to examine the effects of group (control vs. training), time (pre vs. post), and their interaction (group × time) on the dependent variable. Baseline values were included as a covariate to control for initial differences between participants. The model included group, time, and group × time as fixed effects, and subject was treated as a random effect with a random intercept to account for repeated measures. Model parameters were estimated using restricted maximal likelihood (REML), and Type III sums of squares were applied. When appropriate, estimated marginal means were compared using Bonferroni-adjusted pairwise comparisons (Supplementary File S1). For all analyses, the significance level was set at *p* < 0.05. Statistical analyses were performed using SPSS (IBM SPSS Statistics for macOS, Version 24.0; IBM Corp., Armonk, NY, USA). Stability at the end of the test was evaluated using the slope of the linear regression over the final 60 s.

## Results

**Fig. 1:**
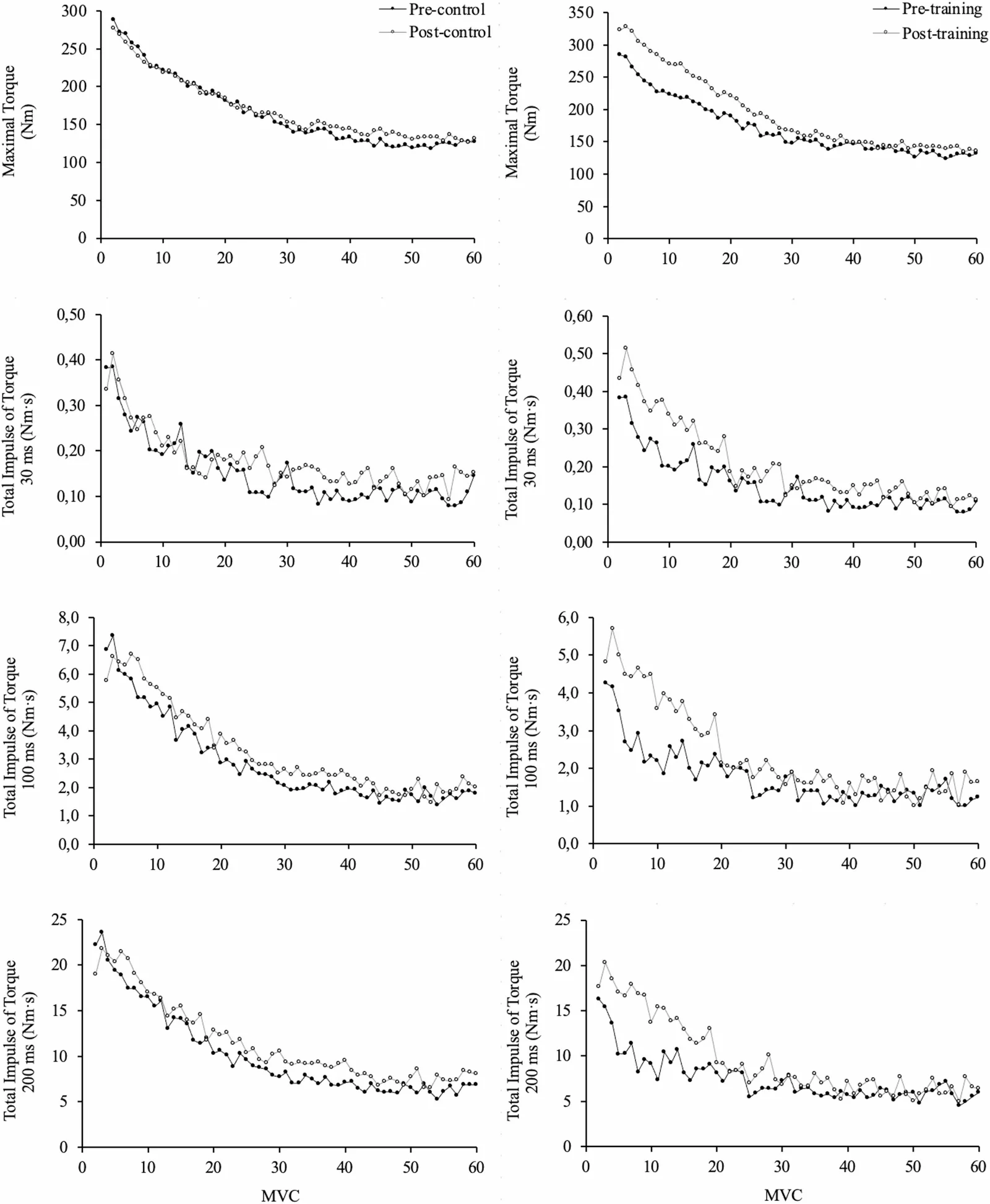
Data are presented as mean maximal torque and total impulse of torque within the 30, 100 and 200 ms time windows, during 60 maximal voluntary contractions (MVC) performed in a 5-min all-out test

Maximal torque during the pre–all-out MVC (non-fatigued condition) was similar to that observed in the first three MVC of the all-out test (all, *p* > 0.05). Both maximal torque and total impulse of torque (30, 100 and 200 ms) progressively declined over the 60 MVC and were well described by an exponential model (*all*, *r* > 0.90, *p* < 0.001; Fig. 1). No significant differences were observed in Δτ between maximal torque and total impulse of torque (*all*, *p* = 0.255) (Table 1). Slopes of the linear regressions for maximal torque and total impulse of torque during the final 60 s did not differ from zero (*all*, −0.03 Nm·s), indicating stabilization at the end of the protocol.

### Non-fatigued muscle

A repeated-measures model applied to the non-fatigued muscle revealed a significant interaction between Group × Variables (ΔMaximal Torque, ΔImpulse 30, 100 and 200 ms) (F_3,51_ = 4.46; *p* = 0.007; ηp² = 0.208). In the univariate analyses, the effect of the Group was significant only for ΔMaximal Torque (F_1,17_ = 4.94; *p* = 0.040; ηp² = 0.225), with no significant differences observed for ΔImpulse 30 ms (*p* = 0.126), ΔImpulse 100 ms (*p* = 0.250), or ΔImpulse 200 ms (*p* = 0.372). The repeated-measures model applied to the iEMG across the 30, 100, and 200 ms windows revealed no significant Group × Variables interaction (F_2,36_ = 0.10; *p* = 0.796; ηp² = 0.006), nor main effects of Group (F_1,18_ = 0.72; *p* = 0.409; ηp² = 0.040) or Variables (F_2,36_ = 1.05; *p* = 0.332; ηp² = 0.058) (Table 2).

### 5-min all-out test

The analysis of Δτ for maximal torque and total impulse of torque (30, 100, and 200 ms) revealed no significant Group × Variable interaction (F_3,51_ = 1.071; *p* = 0.370; ηp² = 0.059). A significant main effect of Group was observed (F_1,17_ = 10.276; *p* = 0.005; ηp² = 0.377), whereas the main effect of Variable did not reach significance (F_3,51_ = 1.333; *p* = 0.274; ηp² = 0.073) (Table 1).

**Fig. 2:**
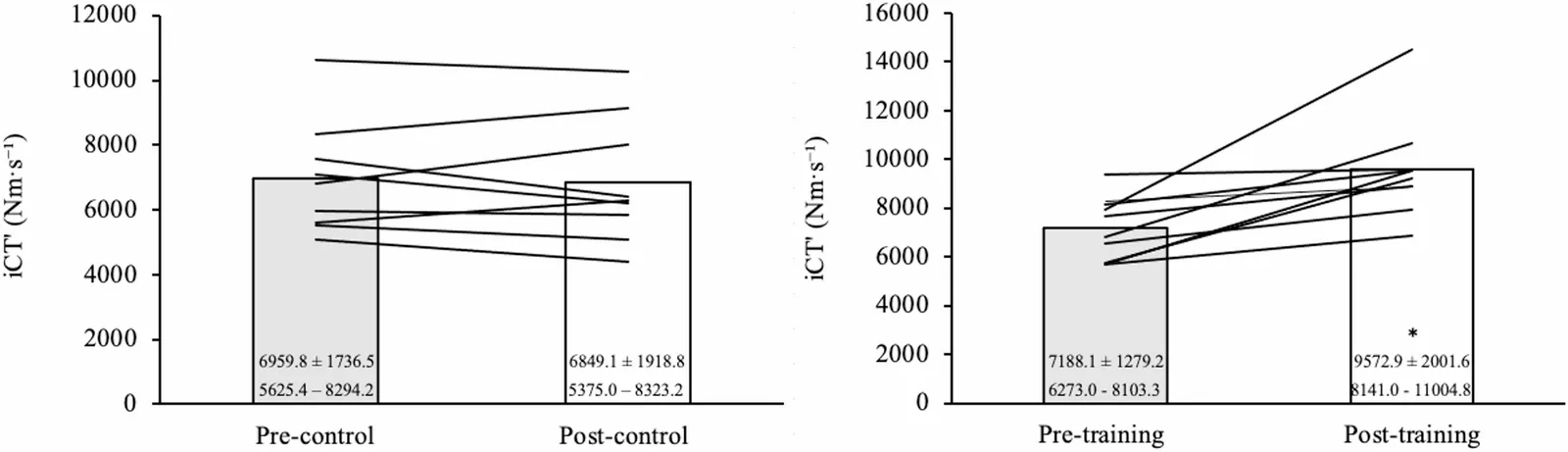
The data are presented as mean ± standard deviation and 95% confidence intervals (95% CI) for the cumulative impulse above CT (iCT’). Bars represent the group mean values, while connecting lines represent individual participant data. *p < 0.05 for comparisons between Pre vs. Post in the Training condition

**Fig. 3:**
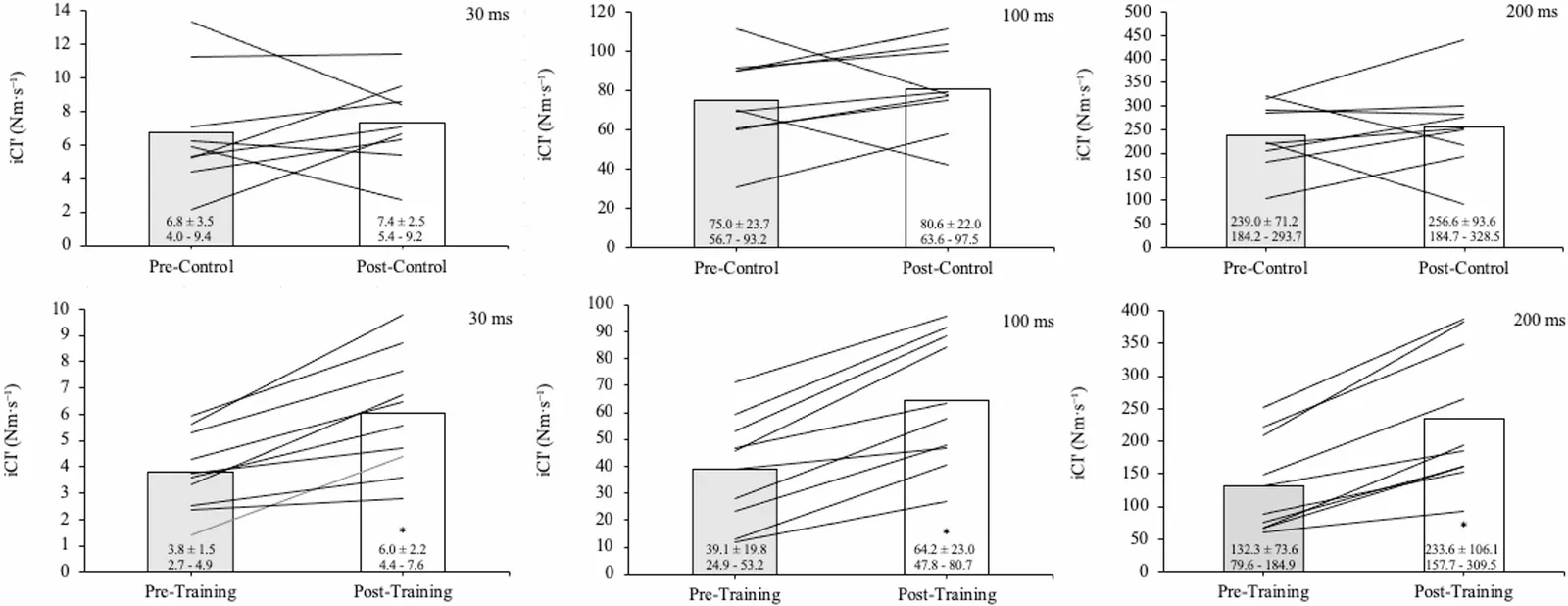
The data are presented as mean ± standard deviation and 95% confidence intervals (95% CI) for the Impulse accumulated above CI (iCI′) in 30, 100, and 200 ms time windows during 60 maximal voluntary contractions (MVC) performed in a 5-min all-out test. Bars represent the group mean values, while connecting lines represent individual participant data. *p < 0.05 for comparisons between Pre vs. Post in the Training condition

Multivariate analyses of the deltas of thresholds relative to maximal values (ΔCT, ΔCI at 30, 100, and 200 ms) showed no significant Group × Variable interaction (F_3,15_ = 0.311; *p* = 0.817; ηp² = 0.059), nor significant main effects of Group (F_1,17_ = 1.992; *p* = 0.176; ηp² = 0.105) or Variable (F_3,15_ = 1.326; *p* = 0.303; ηp² = 0.210) (Table 3). For iCT′ and iCI′ at 30, 100, and 200 ms, no significant Group × Variable interaction was identified (F_3,15_ = 1.349; *p* = 0.269; ηp² = 0.074). However, significant main effects were detected for Group (F_1,17_ = 11.241; *p* = 0.004; ηp² = 0.398) and for Variables (F_3,51_ = 3.335; *p* = 0.026; ηp² = 0.164) (Figs. 2 and 3).

**Fig. 4:**
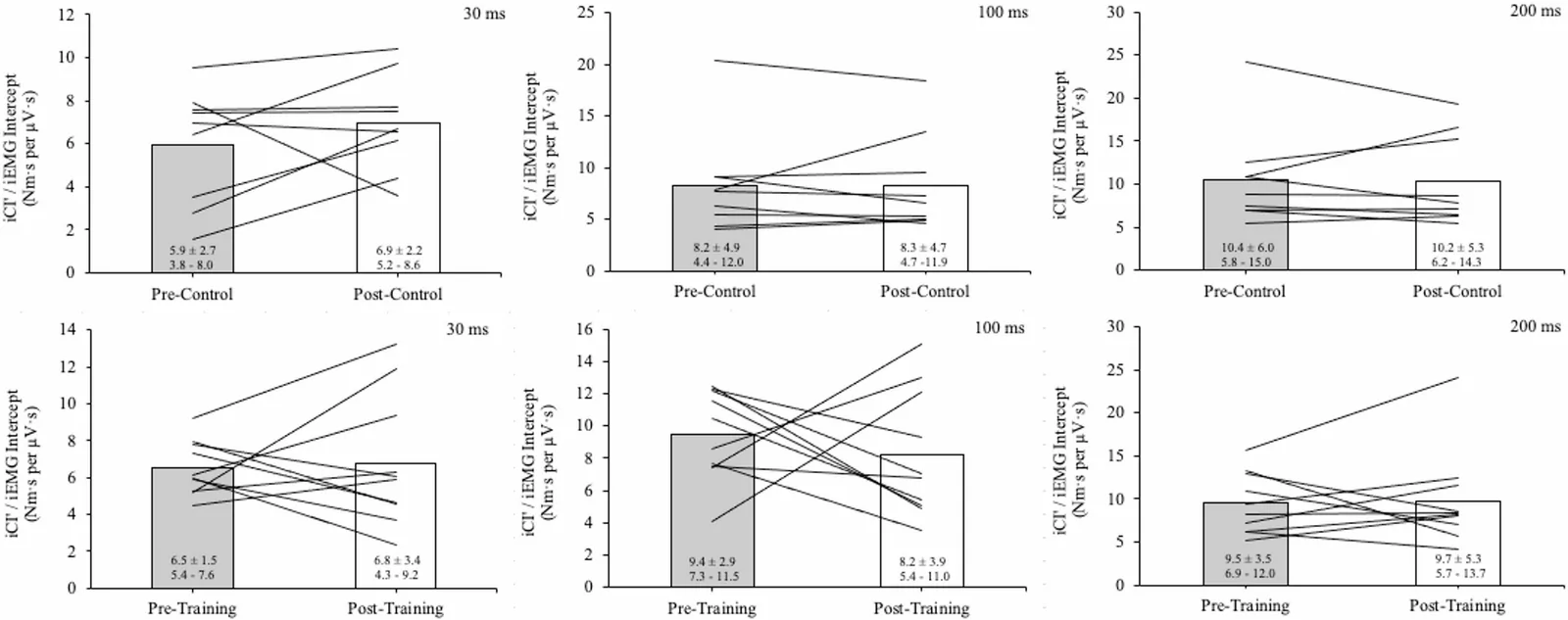
The data are presented as mean ± standard deviation and 95% confidence intervals (95% CI) for the impulse accumulated above CI (iCI′) normalized by the intercept of the integrated electromyography (iEMG) (iCI′/iEMG intercept) in 30, 100, and 200 ms time windows, during 60 maximal voluntary contractions (MVC) performed in a 5-min all-out test. Bars represent the group mean values, while connecting lines represent individual participant data. No significant differences were observed between Pre- and Post-intervention (*p* > 0.05)

The analysis of the iEMG intercept deltas across the 30, 100, and 200 ms windows revealed no significant Group × Variable interaction (F_2,36_ = 2.774; *p* = 0.076; ηp² = 0.134), but significant main effects of Group (F_1,18_= 5.567; *p* = 0.030; ηp² = 0.236) and Variables (F_2,36_ = 6.494; *p* = 0.004; ηp² = 0.265) (Table 4). Although absolute iCI′ values increased following training, normalization by the iEMG intercept removed the significant effect (F_1,17_ = 3.12; *p* = 0.095; ηp² = 0.155) (Fig. 4). Finally, the analysis of the delta iEMG slope revealed no significant Group × Variable interaction (F_2,34_ = 0.546; *p* = 0.584; ηp² = 0.031), nor significant main effects of Group (F_1,17_ = 2.878; *p* = 0.108; ηp² = 0.145) or Variables (F_2,34_ = 1.162; *p* = 0.325; ηp² = 0.064) (Table 4).

**Table 1:** Time constant (τ) decay

		Pre			Post		
		*τ* (s)	95% CI	R^2^	*τ* (s)	95% CI	R^2^
Control	Maximal Torque	73.2 ± 17.8	62.3–84.1	0.98	71.3 ± 17.0	61.3–81.5	0.93
	Impulse 30 ms	75.1 ± 16.4	63.7–87.5	0.96	75.9 ± 16.7	64.3–88.3	0.94
	Impulse 100 ms	69.6 ± 19.1	55.6–85.6	0.92	73.3 ± 19.5	58.8–88.6	0.95
	Impulse 200 ms	73.7 ± 18.0	61.6–88.0	0.94	71.7 ± 18.0	59.8–86.2	0.91
Training	Maximal Torque	57.0 ± 13.1	49.1–64.4	0.97	79.8 ± 18.0*	67.6–92.3	0.97
	Impulse 30 ms	61.6 ± 15.4	51.6–72.0	0.91	73.3 ± 14.8*	64.6–83.0	0.91
	Impulse 100 ms	56.8 ± 11.3	50.8–60.8	0.92	76.1 ± 19.5*	66.9–89.1	0.93
	Impulse 200 ms	55.2 ± 13.8	47.9–62.5	0.94	84.0 ± 17.3*	73.7–96.3	0.93

Data are presented as mean ± standard deviation and 95% confidence intervals (95% CI). The τ values (in seconds) and the respective coefficients of determination (R²) for the exponential fits of maximal torque and total impulse of torque at 30, 100, and 200 ms during the 5-min all-out test. *p < 0.05 for comparisons between Pre vs. Post in the Training condition

**Table 2:** Maximal Torque, Torque Impulse, and Integrated Electromyography (iEMG) during Maximal Voluntary Contraction (MVC) in Non-Fatigued Muscle

			Control				Training			
Variables		Units	Pre	95% CI	Post	95% CI	Pre	95% CI	Post	95% CI
Maximal Torque		Nm	300.1 ± 70.0	252.2 - 348.1	317.8 ± 66.0	270.9 - 347.7	301.6 ± 51.6	270.9 - 330.4	347.2 ± 45.3*	306.7 - 385.8
30 ms	IImpulse	Nm•s	0.39 ± 0.08	0.33 - 0.46	0.40 ± 0.09	0.33 - 0.47	0.39 ± 0.18	0.27 - 0.50	0.41 ± 0.20	0.28 - 0.53
	iEMG	µV•s	1.5 ± 0.7	0.6 - 2.3	1.3 ± 0.9	0.3 - 2.3	1.0 ± 1.0	0.2 - 2.1	1.0 ± 0.8	1.0 ± 0.8
100 ms	IImpulse	Nm•s	6.8 ± 2.6	4.8 - 8.8	6.2 ± 1.8	4.7 - 7.6	4.4 ± 2.0	2.9 - 6.0	4.7 ± 1.8	3.2 - 6.1
	iEMG	µV•s	14.2 ± 7.6	8.0 - 20.4	14.0 ± 8.7	6.3 - 21.6	8.5 ± 3.6	5.9 - 11.2	9.7 ± 7.3	3.3 - 16.0
200 ms	IImpulse	Nm•s	21.8 ± 7.6	16.0 – 27.7	19.6 ± 6.1	14.9 - 24.4	16.1 ± 6.3	11.1 - 21.2	17.3 ± 6.9	11.9 - 22.6
	iEMG	µV•s	29.5 ± 14.1	19.1 - 39.8	28.9 ± 17.4	17.8 - 40.0	24.8 ± 16.7	11.1 - 21.2	25.8 ± 16.8	12.7 - 38.9

Data are presented as mean ± standard deviation and 95% confidence intervals (95% CI). *p < 0.05 compared with Pre within the same group (Training) for maximal torque

**Table 3:** Critical torque (CT) and critical impulse (CI)

			Control			Training			
Variables	Window duration & Variable units	Pre	95% CI	Post	95% CI	Pre	95% CI	Post	95% CI
CT	Nm	94.8 ± 18.4	80.6–108.8	96.2 ± 31.3	72.2–120.1	100.3 ± 26.6	85.6–114.5	100.4 ± 17.3	90.2–110.2
	%	34.0 ± 8.1	28.6–40.8	34.6 ± 6.7	29.9–38.9	34.3 ± 7.5	28.9–40.0	30.2 ± 5.3	25.9–33.8
CI	30 ms								
	Nm·s	0.15 ± 0.09	0.06–0.21	0.13 ± 0.04	0.09–0.18	0.10 ± 0.05	0.08–0.12	0.11 ± 0.08	0.09–0.14
	%	28.5 ± 19.8	13.8–43.2	31.3 ± 18.9	21.8–40.9	29.9 ± 8.70	26.1–35.6	29.6 ± 5.79	26.2–34.5
	100 ms								
	Nm·s	1.78 ± 1.05	0.67–2.90	1.96 ± 1.06	0.82–3.11	1.31 ± 0.69	0.63–1.98	1.48 ± 0.88	0.70–2.26
	%	28.6 ± 20.0	13.2–43.0	31.7 ± 18.4	16.7–46.8	29.7 ± 6.59	25.0–34.4	30.6 ± 4.73	27.7–33.6
	200 ms								
	Nm·s	6.64 ± 3.05	3.85–9.43	6.70 ± 2.87	3.94–9.47	5.87 ± 3.20	3.66–8.08	6.19 ± 3.57	3.67–8.72
	%	31.6 ± 17.8	14.5–48.7	32.9 ± 14.3	22.4–43.2	31.9 ± 11.1	23.1–40.6	29.9 ± 9.13	22.9–36.9

Data are presented as mean ± standard deviation and 95% confidence intervals (95% CI). No significant differences were observed between Pre- and Post-intervention (p> 0.05)

## Discussion

The performance analysis of the 5-min all-out test confirmed the validity of the hyperbolic RTD–time model (Abdalla et al. 2025), demonstrating its sensitivity to detect training-induced adaptations specific to maximal strength when quantified through mechanical impulse. The findings of the present study fully support the proposed hypotheses. In non-fatigued muscle, (i) training significantly increased maximal torque without producing consistent changes in impulse (30, 100, and 200 ms) or in the associated neural activity (iEMG). During the 5-min all-out test, (ii) training enhanced the ability to sustain maximal and rapid torque production under fatigue via a slower decline in the τ, indicating greater fatigue resistance; (iii) furthermore, training increased iCT′ and iCI′ without altering the CT and CI across all time windows.

**Table 4:** Integrated electromyography (iEMG) activation parameters

		Control				Training			
Window duration	Parameters	Pre	95% CI	Post	95% CI	Pre	95% CI	Post	95% CI
30 ms	Intercept (µV·s)	1.47 ± 1.30	0.43–2.52	1.16 ± 0.54	0.71–1.60	0.61 ± 0.30	0.38–0.84	1.05 ± 0.49*	0.73–1.48
	Slope (µV·s/ms)	−0.0059 ± 0.0069	−0.0116–0.0002	−0.0062 ± 0.0087	−0.0139–0.0015	−0.0048 ± 0.0064	−0.0010–0.0106	−0.0023 ± 0.0014	−0.0132–0.0086
100 ms	Intercept (µV·s)	10.9 ± 6.2	5.84–16.1	11.7 ± 5.1	7.96–15.5	4.28 ± 2.11	2.66–5.94	9.72 ± 5.73*	5.06–14.3
	Slope (µV·s/ms)	−0.0807 ± 0.0551	−0.178–0.012	−0.0826 ± 0.0862	−0.189–0.024	−0.0708 ± 0.0440	−0.0166–0.1249	−0.0220 ± 0.0680	−0.0495–0.0491
200 ms	Intercept (µV·s)	26.1 ± 11.4	16.5–35.8	28.6 ± 12.9	17.6–39.5	14.9 ± 9.7	8.19–21.6	28.5 ± 16.0*	16.1–41.0
	Slope (µV·s/ms)	−0.1136 ± 0.1363	−0.309–0.082	−0.1594 ± 0.2323	−0.406–0.087	−0.1931 ± 0.126	−0.027–0.359	−0.0124 ± 0.207	−0.099–0.074

Data are presented as mean ± standard deviation and 95% confidence intervals (95% CI) for parameters derived from the linear regression of integrated electromyography (iEMG) across 60 maximal voluntary contractions (MVC) during a 5-min all-out test (intercept and slope). *p < 0.05 for comparisons between Pre vs. Post in the Training condition

### Non-fatigued muscle

After three weeks of intervention, the control group showed no significant changes in any measured variable. In contrast, the training group exhibited a significant increase in maximal torque in non-fatigued muscle, without consistent changes in torque impulse or iEMG within the 30, 100, and 200 ms windows during MVC. These findings suggest that training induced neural adaptations specific to sustained force production, without altering the capacity for rapid torque generation (Aagaard et al. 2002; Maffiuletti et al. 2016).

The magnitude of muscle activation is controlled by two central neural components: (i) the number of recruited motor units, defined as the ensemble of a motoneuron and the muscle fibers it innervates, and (ii) the firing rate of these units (Duchateau and Enoka 2011; Heckman and Enoka 2012). Changes in either determinant directly modulate the temporal profile of force and, consequently, the RTD and impulse (Aagaard et al. 2002). According to the literature, training-induced adaptations are restricted to the temporal and functional domain of the applied stimulus (Maffiuletti et al. 2016). Maximal strength protocols tend to enhance the maintenance of neural drive and sustained voluntary force (Bassan et al. 2019; Del Vecchio et al. 2023), whereas gains in RTD and impulse require stimuli capable of modifying early neural behavior, including recruitment speed and motor unit firing rate (Aagaard et al. 2002). For instance, Del Vecchio et al. (2023) reported that increases in maximal torque following isometric training are associated with elevated motor unit firing rates during the late contraction phase (~ 300 ms), reflecting intensified sustained neural drive. In contrast, the early contraction phase (< 200 ms), which underlies rapid force development, showed no changes in recruitment speed or initial motor unit discharge, and consequently, no increase in RTD. These findings account for the observed stability in both impulse and iEMG across the 30, 100, and 200 ms windows in the present study, supporting the notion that rapid torque production is regulated by neural circuits distinct from those mediating maximal force.

### 5-min all-out test

After three weeks of intervention, the control group showed no significant changes in any of the measured variables. In contrast, the assessment of maximal torque kinetics and total impulse of torque (30, 100, and 200 ms) in the Training group revealed a consistent increase in τ, indicating a slower neuromechanical decline over the 60 contractions.

Mathematically, a higher τ, derived from the initial amplitude relative to the asymptotic value, implies that maximal torque and total impulse of torque decrease more gradually. Given that there were no changes in the critical limits (CT and CI), this deceleration results in an increased area under the curve, suggesting an enhancement of iCT′ and iCI′. This finding is particularly relevant considering the metabolic kinetics of the 5-min all-out protocol in knee extensors (3 s on–2 s off), especially during the early stages of the test. As demonstrated by Burnley et al. (2009), by approximately 120 s, there is already a ~ 35–40% loss of peripheral contractile capacity, indicating that a significant portion of excitation–contraction coupling is compromised. Subsequent evidence obtained via magnetic resonance spectroscopy (Burnley et al. 2010; Broxterman et al. 2018) supports this interpretation, showing that by the 20th MVC (~ 120 s), approximately 70–75% of the total phosphocreatine (PCr) depletion, 75–85% of inorganic phosphate (Pi) accumulation, 65–80% of dihydrogen phosphate (H₂PO₄⁻) elevation, and 60–70% of proton (H⁺) accumulation have already occurred, characterizing this interval as the phase of greatest bioenergetic disturbance. Consistently, in the present study, we observed that approximately ~ 80% of iCT′ and iCI′ were consumed during this same period (120 s), reinforcing that subsequent performance critically depends on the ability to resist the initial metabolic deterioration during the 5-min all-out test. Therefore, we can suggest that the increase in iCT′ and iCI′ observed in this study is consistent with a reduced functional impact of the rapid accumulation of metabolites (Pi, H₂PO₄⁻, H⁺) during the early phase of the test. Reduced bioenergetic perturbation during this period would diminish activation of group III/IV afferents, which are known to be sensitive to these metabolic changes (Amann et al. 2009; Hureau et al. 2016; Broxterman et al. 2018). Although Bassan et al. (2019) did not directly assess afferent activity, they reported increased W′ and greater tolerance to peripheral fatigue following similar maximal strength training, a functional pattern consistent with this interpretation.

The observed increases in τ and iCI′ in the three windows (30, 100, and 200 ms) combined with the absence of interaction, indicate that the entire temporal spectrum of torque development responded consistently to training. Although the first 30 ms are classically described as being predominantly determined by neural drive (Aagaard et al. 2002; de Ruiter et al. 2004) and relatively insensitive to metabolic perturbations (Marshall et al. 2015; Varesco et al. 2022), the present findings suggest that peripheral adaptations were sufficient to preserve contractile capacity from the very onset of contraction. This early preservation aligns with the physiological profile that characterizes the first 120 s of the all-out test, a period in which tolerance to intense bioenergetic disturbance is crucial for maintaining torque output (Burnley et al. 2010; Broxterman et al. 2017). During this interval, the muscle relies on mechanisms that attenuate the impact of acidosis, PCr depletion, and metabolite accumulation (Hureau et al. 2016). Among these mechanisms, efficient Ca²⁺ handling, actin–myosin kinetics, and modulation of group III/IV afferent feedback play central roles, all of which are well known to be influenced by peripheral fatigue (Westerblad and Allen 1991; Fitts 2008; Amann et al. 2009). Although these processes are traditionally associated with later phases of force development (Westerblad and Allen 1991; Fitts 2008), evidence from the present study indicates that they may be recruited from the very onset of contraction under conditions of elevated metabolic demand, contributing not only to the maintenance of force during sustained efforts but also to initial performance in very short time windows. Accordingly, the parallel increases observed across all temporal windows appear to reflect a global neuromuscular adaptation to cope with the acute metabolic stress that characterizes the onset of an all-out effort. This continuous perspective reveals aspects of contractile resilience that are not captured by isolated post-fatigue MVC measures, reinforcing the sensitivity of the iCI′ to detect early alterations in rapid force-generating capacity.

A detailed analysis of iEMG within the (30, 100, and 200 ms) windows across the 60 contractions revealed a consistent neural pattern aligned with the mechanical adaptations observed for τ and iCI′. The central finding was the consistent increase in the iEMG intercept following training, indicating that, during maximal effort (all-out), participants initiated each contraction with a higher absolute level of neural activation. This interpretation is supported by the stability of the iCI′ normalized by the intercept (iCI′/intercept), suggesting that the increase in intercept reflected greater neural activation, facilitated by a reduction in contractile decay (Burnley et al. 2010; Broxterman et al. 2017). These findings are consistent with the literature suggesting that training may have improved peripheral fatigue tolerance, resulting in less force degradation (Bassan et al. 2019). The increase in iEMG intercept may reflect neural adaptations such as enhanced motor unit firing rates, improved recruitment strategies, or reduced antagonist co-contraction, as highlighted in recent reviews on EMG interpretation (Lecce et al. 2026). In addition, a reduced influence of inhibitory afferent feedback (group III/IV) is also a plausible contributing mechanism, particularly in the context of improved peripheral fatigue tolerance (Hureau et al. 2016; Broxterman et al. 2018; Bassan et al. 2019). This interpretation is consistent with previous evidence linking metabolic perturbation to afferent-mediated inhibition of central motor drive (Amann et al. 2009; Hureau et al. 2016; Broxterman et al. 2018). However, as no direct measurements of afferent activity were obtained, this mechanism should be interpreted with caution. Importantly, this increase in activation occurred without changes in the slope coefficient of the iEMG–5-min all-out test relationship, indicating that training did not influence the dynamics of neural drive decline during fatigue. In other words, while muscles became more resistant to peripheral fatigue, the central mechanisms responsible for controlling the decline in neural command in response to fatigue remained unchanged.

### Theoretical and practical implications

The present study represents an important advance in modeling the capacity for rapid torque production under fatigue, refining and critically testing the model proposed by Abdalla et al. (2025), which is based on the hyperbolic RTD-time relationship, derived from the slope of the torque-time curve. By incorporating the concept of torque impulse, a cumulative and integrable measure, this study overcomes the limitations of slope-based models, providing a more comprehensive and physiologically consistent representation of rapid neuromuscular capacity. Measures based on impulse (the integral of the force-time curve) accumulate variations of the discrete instantaneous derivative throughout the ascending phase of the contraction, reflecting both the initial and sequential recruitment of motor units, and the subsequent increase in neural firing rate (Maffiuletti et al. 2016), in accordance with the classical force-control models proposed by Duchateau and Enoka (2011) and Heckman and Enoka (2012). Moreover, this approach demonstrates greater sensitivity to neuromuscular adaptations than instantaneous estimates of slope or peak RTD (Chatrenet et al. 2023). This demonstration underscores the conceptual robustness and physiological validity of the hyperbolic impulse-time model, highlighting the dissociation between two functionally complementary components of rapid torque production: CI and iCI′.

## Conclusion

Maximal strength training increased maximal torque in the non-fatigued condition, without changes in torque impulse or early electromyographic activity. During the 5-min all-out test, training slowed the decline in torque and torque impulse, increasing iCT′ and iCI′ without modifying CT or CI. These results indicate that training improves fatigue tolerance and the ability to rapidly produce torque under fatigue, without altering the critical limits of the neuromuscular system.

## Supplementary Information

Below is the link to the electronic supplementary material.

Supplementary Material 1

## Data Availability

The data that support the findings of this study are available from the corresponding author upon reasonable request.

## Abbreviations

CI: Critical impulse
CP: Critical power
CT: Critical torque
EMG: Electromyography
iCI′: Impulse accumulated above CI
iCT′: Impulse accumulated above CT
iEMG: Integrated electromyography
MVC: Maximal voluntary contraction
RTD: Rate of torque development

## List of symbols

τ: Time constant
W′: Capacity for work above CP
