# Correction: An integrated model of rapid torque production: neuromuscular adaptations under fatigue after strength training

**DOI:** 10.1007/s00421-026-06353-z

**Published:** 2026-09-02

**Authors:** Leonardo Henrique Perinotto Abdalla, Benedito Sérgio Denadai, Natália de Menezes Bassan, Ryan Michael Broxterman, Camila Coelho Greco

**Affiliations:** 1 Human Performance Laboratory, UNESP, Av. 24 A, 1515, Bela Vista, Rio Claro, S.P CEP – 13506-900 Brazil; 2 Department of Internal Medicine, University of Utah, Salt Lake, UT USA; 3 Geriatric Research, Education, and Clinical Center, VAMC, Salt Lake, UT USA

**Correction: European Journal of Applied Physiology** https://doi.org/10.1007/s00421-026-06321-7

In the original version of this article, table 4 was duplicated as table 2 and table 2 was missing.

The correct Table 2 should have appeared as shown below.

**Table 2:** Maximal Torque, Torque Impulse, and Integrated Electromyography (iEMG) during Maximal Voluntary Contraction (MVC) in Non-Fatigued Muscle

			Control				Training			
Variables		Units	Pre	95% CI	Post	95% CI	Pre	95% CI	Post	95% CI
Maximal Torque		Nm	300.1 ± 70.0	252.2–348.1	317.8 ± 66.0	270.9–347.7	301.6 ± 51.6	270.9–330.4	347.2 ± 45.3*	306.7–385.8
30 ms	Impulse	Nm·s	0.39 ± 0.08	0.33–0.46	0.40 ± 0.09	0.33–0.47	0.39 ± 0.18	0.27–0.50	0.41 ± 0.20	0.28–0.53
	iEMG	µV·s	1.5 ± 0.7	0.6–2.3	1.3 ± 0.9	0.3–2.3	1.0 ± 1.0	0.2–2.1	1.0 ± 0.8	0.1–1.9
100 ms	Impulse	Nm·s	6.8 ± 2.6	4.8–8.8	6.2 ± 1.8	4.7–7.6	4.4 ± 2.0	2.9–6.0	4.7 ± 1.8	3.2–6.1
	iEMG	µV·s	14.2 ± 7.6	8.0–20.4	14.0 ± 8.7	6.3–21.6	8.5 ± 3.6	5.9–11.2	9.7 ± 7.3	3.3–16.0
200 ms	Impulse	Nm·s	21.8 ± 7.6	16.0–27.7	19.6 ± 6.1	14.9–24.4	16.1 ± 6.3	11.1–21.2	17.3 ± 6.9	11.9–22.6
	iEMG	µV·s	29.5 ± 14.1	19.1–39.8	28.9 ± 17.4	17.8–40.0	24.8 ± 16.7	11.8–37.9	25.8 ± 16.8	12.7–38.9

Data are presented as mean ± standard deviation and 95% confidence intervals (95% CI). *p < 0.05 compared with Pre within the same group (Training) for maximal torque.

The original article has been corrected.

