# Supplementary material for: An integrated model of rapid torque production: neuromuscular adaptations under fatigue after strength training
Source: Eur J Appl Physiol. 2026 Jul 2;126(10):5437–48. doi: 10.1007/s00421-026-06321-7 (PMC13634015; doi:10.1007/s00421-026-06321-7)
Supplement: Supplementary file 1 [file 421_2026_6321_MOESM1_ESM.docx]

**Results of the linear mixed-effects model for absolute values (pre- vs. post-intervention)**

**Non-Fatigued Muscle**

### **Maximal Torque**

A linear mixed-effects model revealed a significant group × time interaction for maximal torque (F(1,33) = 4.581, p = 0.040), indicating a differential training effect over time between groups. Significant main effects were also observed for group (F(1,33) = 4.618, p = 0.039) and time (F(1,33) = 4.234, p = 0.048).

### **Impulse at 30, 100, and 200 ms**

A linear mixed-effects model was applied to impulse at 30, 100, and 200 ms. No significant group × time interaction was observed (30 ms: F(1,33) = 0.063, p = 0.803; 100 ms: F(1,33) = 2.056, p = 0.161; 200 ms: F(1,33) = 1.665, p = 0.206), nor were there significant main effects of group (30 ms: F(1,33) = 0.035, p = 0.853; 100 ms: F(1,33) = 0.001, p = 0.977; 200 ms: F(1,33) = 0.125, p = 0.726) or time (30 ms: F(1,33) = 0.190, p = 0.666; 100 ms: F(1,33) = 0.472, p = 0.497; 200 ms: F(1,33) = 0.160, p = 0.691).

### **iEMG**

A linear mixed-effects model was applied to iEMG during MVC at 30, 100, and 200 ms. No significant group × time interaction was found (30 ms: F(1,33) = 0.381, p = 0.541; 100 ms: F(1,33) = 0.163, p = 0.689; 200 ms: F(1,33) = 0.060, p = 0.808), nor were there significant main effects of group (30 ms: F(1,33) = 0.001, p = 0.970; 100 ms: F(1,33) = 0.017, p = 0.898; 200 ms: F(1,33) = 0.002, p = 0.966) or time (30 ms: F(1,33) = 0.098, p = 0.756; 100 ms: F(1,33) = 0.081, p = 0.778; 200 ms: F(1,33) = 0.004, p = 0.949).

**5-min All-Out Test**

### **Critical Torque (absolute and relative)**

A linear mixed-effects model, with group and time as fixed factors and baseline as a covariate, was applied to absolute and relative critical torque. The group × time interaction was not significant (absolute: F(1,33) = 0.022, p = 0.884; relative: F(1,33) = 2.572, p = 0.118), nor were the main effects of group (absolute: F(1,33) = 0.015, p = 0.904; relative: F(1,33) = 2.367, p = 0.133) and time (absolute: F(1,33) = 0.010, p = 0.920; relative: F(1,33) = 1.444, p = 0.238).

### **Absolute Critical Impulse (30, 100, and 200 ms)**

Absolute critical impulse was analyzed using linear mixed-effects models. No significant group × time interaction was observed (30 ms: F(1,33) = 0.924, p = 0.344; 100 ms: F(1,33) = 0.001, p = 0.981; 200 ms: F(1,33) = 0.047, p = 0.830). Similarly, no significant main effects of group or time were found.

### **Relative Critical Impulse (30, 100, and 200 ms)**

There was **no significant group × time interaction** (30 ms: F(1,33) = 0.228, p = 0.636; 100 ms: F(1,17) = 0.505, p = 0.487; 200 ms: F(1,17) = 0.505, p = 0.487), indicating that changes over time did not differ between groups. Likewise, there was **no main effect of group** (30 ms: F(1,33) = 0.123, p = 0.728; 100 ms: F(1,16) = 0.413, p = 0.529; 200 ms: F(1,33) = 0.734, p = 0.398) or **time** (30 ms: F(1,33) = 0.153, p = 0.698; 100 ms: F(1,17) = 1.424, p = 0.249; 200 ms: F(1,33) = 0.044, p = 0.834).

### **iCT′ and iCI′ (30, 100, and 200 ms)**

Linear mixed-effects models showed a significant group × time interaction for iCT′ (F(1,17) = 12.303, p = 0.003) and for iCI′ at 100 ms and 200 ms (both F(1,17) = 7.286, p = 0.015), but not at 30 ms (F(1,33) = 2.660, p = 0.112). Significant main effects of group and time were also observed for most outcomes.

### **iCI′ Normalized by iEMG**

There were no significant group × time interactions for any of the time intervals (30 ms: F(1,33) = 0.270, p = 0.607; 100 ms: F(1,33) = 0.426, p = 0.519; 200 ms: F(1,33) = 0.041, p = 0.842), indicating that changes over time were similar between groups. Likewise, no main effects of group or time were observed across conditions (30 ms: group: F(1,33) = 0.083, p = 0.775; time: F(1,33) = 0.848, p = 0.364; 100 ms: group: F(1,33) = 0.097, p = 0.758; time: F(1,33) = 0.308, p = 0.583; 200 ms group: F(1,33) = 0.011, p = 0.917; 200 ms time: F(1,33) = 0.003, p = 0.997).

### **iEMG Slope (30, 100, and 200 ms)**

There were no significant group × time interactions for any of the time intervals (30 ms: F(1,33) = 3.186, p = 0.083; 100 ms: F(1,33) = 3.386, p = 0.075; 200 ms: F(1,33) = 3.086, p = 0.095), indicating that changes over time were similar between groups. Likewise, no main effects of group were observed (30 ms: F(1,33) = 0.433, p = 0.515; 100 ms: F(1,33) = 0.663, p = 0.421; 200 ms: F(1,33) = 0.45, p = 0.505), and no significant main effects of time were found across conditions (30 ms: F(1,33) = 3.777, p = 0.061; 100 ms: F(1,33) = 3.339, p = 0.077; 200 ms: F(1,33) = 3.386, p = 0.075).

### **iEMG Intercept (30, 100, and 200 ms)**

A significant group × time interaction was observed at 100 ms (F(1,33) = 4.632, p = 0.039) and 200 ms (F(1,17) = 4.503, p = 0.049), but not at 30 ms. No significant main effect of group was observed, while time showed significance at 100 and 200 ms only.

### **Tau (τ) for Maximal Torque and Impulse (30, 100, and 200 ms)**

There were **significant group × time interactions** for all variables (Maximal torque: F(1,33) = 122.627, p < 0.001; 30 ms: F(1,33) = 333.524, p < 0.001; 100 ms: F(1,33) = 45.071, p < 0.001; 200 ms: F(1,33) = 390.580, p < 0.001), indicating differential changes between groups over time. In addition, **significant main effects** of **group** (Maximal torque: F(1,33) = 103.606, p < 0.001; 30 ms: F(1,33) = 231.178, p < 0.001; 100 ms: F(1,33) = 46.063, p < 0.001; 200 ms: F(1,33) = 307.581, p < 0.001) and **time** (Maximal torque: F(1,33) = 90.200, p < 0.001; 30 ms: F(1,33) = 432.394, p < 0.001; 100 ms: F(1,33) = 70.139, p < 0.001; 200 ms: F(1,33) = 298.500, p < 0.001 ) were observed.

| Control | | | | Training | |
| --- | --- | --- | --- | --- | --- |
| Parameters | | Mean±SD | 95% CI | Mean±SD | 95% CI |
| MVC | Max Torque | 0.6± 8.1 | -5.28 to 6.4 | 16.7± 20.8* | 1.93 to 31.5 |
|  | Impulse 30 ms | 4.5± 27.0 | 14.97 to 24.0 | 18.4± 44.7 | 12.79 to 49.7 |
|  | Impulse 100 ms | 9.5± 67.5 | -44.33 to 63.3 | 10.4± 28.7 | -9.98 to 30.9 |
|  | Impulso 200 ms | 3.8± 55.2 | -39.08 to 46.9 | 14.2± 49.8 | -21.24 to 49.7 |
|  | iEMG 30 ms | 12.8± 103.6 | 68.36 to 92.7 | 24.9± 69.1 | -28.12 to 69.1 |
|  | iEMG 100 ms | 15.4± 67.9 | -37.10 to 67.8 | 5.3± 52.9 | 33.15 to 43.7 |
|  | iEMG 200 ms | 17.5± 85.1 | -44.90 to 79.8 | 18.4± 60.2 | -25.89 to 62.7 |
| Tau | Max Torque | -2.3± 2.8 | 4.40 to -0.15 | 40.2± 4.4* | 37.1 to 43.4 |
|  | 30 ms | 1.0± 1.5 | -0.6 to -2.2 | 20.8± 7.5* | 15.2 to 26.4 |
|  | 100 ms | 3.8± 1.6 | 2.2 to -5.0 | 38.4±10.1* | 30.5 to 46.3 |
|  | 200 ms | -2.8± 0.6 | -3.26 to -1.7 | 54.6± 4.7* | 51.2 to 58.0 |
| CT | abs | 1.2± 19.7 | -14.16 to 16.0 | 5.7± 27.6 | -12.20 to 23.6 |
|  | % | 5.3± 23.6 | -13.19 to 23.0 | -10.4± 17.9 | -22.75 to 2.05 |
| CI | abs 30 ms | -2.7± 45.9 | -42.53 to 100.7 | 32.5± 81.5 | -24.28 to 89.3 |
|  | % 30 ms | 21.3± 79.8 | -43.20 to 162.3 | 7.9± 38.1 | -16.46 to 32.3 |
|  | abs 100 ms | 23.1± 56.1 | -22.35 to 68.3 | 14.7± 49.5 | -20.26 to 77.1 |
|  | % 100 ms | 13.7± 28.3 | -7.89 to 35.2 | 9.5± 38.0 | -15.90 to 34.9 |
|  | abs 200 ms | 12.2± 39.7 | -18.39 to 42.7 | 16.4± 47.6 | -15.38 to 48.3 |
|  | % 200 ms | 7.8± 37.3 | -23.47 to 70.3 | -1.0± 23.6 | -16.71 to 13.8 |
| iCT'  iCI' | abs | -1.7± 12.2 | -10.89 to 7.43 | 35.3± 28.5* | 15.16 to 55.5 |
|  | 30 ms | 33.1± 81.3 | -33.62 to 99.7 | 67.3± 56.2* | 27.45 to 107.1 |
|  | 100 ms | 14.8± 36.3 | -13.16 to 42.6 | 84.6± 57.6* | 46.35 to 122.8 |
|  | 200 ms | 13.8± 43.0 | -19.92 to 47.4 | 88.8± 47.6* | 56.37 to 121.1 |
| iEMG Intercept | 30 ms | 3.3± 51.9 | -36.60 to 43.2 | 110.1± 138.9* | 10.80 to 209.4 |
|  | 100 ms | 18.9± 46.8 | -17.10 to 54.9 | 166.0± 179.8* | 37.40 to 294.6 |
|  | 200 ms | 16.4± 43.2 | -16.83 to 49.6 | 119.4± 142.5* | 17.50 to 221.3 |
| iEMG Slope | 30 ms | -40.3± 197.1 | -186.9 to 106.1 | -202.1± 320.7 | -412.9 to 7.5 |
|  | 100 ms | -35.8± 141.5 | -144.80 to 73.2 | -102.8± 136.1 | -200.0 to -5.4 |
|  | 200 ms | 6.9± 105.9 | -74.52 to 88.2 | -84.7± 134.9 | -181.1 to 11.8 |
| iCI' / iEMG Intercept | 30 ms | 45.2± 77.6 | 14.15 to 104.5 | 7.8± 59.0 | -34.34 to 50.0 |
|  | 100 ms | 4.7± 30.2 | -20.29 to 28.7 | 5.5± 85.2 | -55.53 to 66.4 |
|  | 200 ms | 0.7± 26.4 | 19.54 to 21.0 | 7.5± 45.3 | -24.90 to 39.8 |

Table S1. Values are presented as mean ± standard deviation (SD), with 95% confidence intervals (95% CI) reported for the deltas (Δ). Asterisks (*) denote statistically significant differences compared to the Training condition (p < 0.05). The table reports variables derived from maximal voluntary contraction (MVC), including maximal torque, as well as torque–time impulse and integrated electromyography (iEMG), which were calculated over 30, 100, and 200 ms time windows. It also includes variables derived from the 5-min all-out test, such as the decay time constant (τ) of maximal torque and total impulse over 30, 100, and 200 ms windows. Additional measures include critical torque (CT) and critical impulse (CI; 30, 100, and 200 ms), expressed in both absolute and relative terms, as well as impulse above CT (iCT’) and impulse above CI (iCI’) across the same time windows. Additionally, normalized iCI’ (with respect to the iEMG intercept), as well as the iEMG intercept and slope, are reported.
